# Monitoring Method of Total Seed Mass in a Vibrating Tray Using Artificial Neural Network

**DOI:** 10.3390/s18113659

**Published:** 2018-10-28

**Authors:** Zhan Zhao, Fang Qin, Chun-Jie Tian, Simon X. Yang

**Affiliations:** 1 Institute of Agriculture Engineering, Jiangsu University, Zhenjiang 212013, China; qinfang0421@163.com; 2 School of Engineering, University of Guelph, Guelph, ON N1G 2W1, Canada; tianchunjie1509@163.com (C.-J.T.); syang@uoguelph.ca (S.X.Y.)

**Keywords:** vacuum plate seeder, total seed mass, monitoring method, rectangular vibrating tray, discrete element method, neural network

## Abstract

To maintain the continuous working performance of a vacuum plate seeder, it is important to monitor the total seed mass in the seed tray in real time and accurately control the pickup position of the suction plate accordingly. Under the excitation of reciprocating vibration varying with time and interference by direction angle, the motion of seeds in a rectangular tray was simulated using the discrete element method (DEM). A measurement method for seed mass in a small area was proposed based on the impulse theorem. The impact force of seeds was monitored with a cantilever force sensor, and the corresponding signal processing circuit was designed. Calibration results indicated that the relative nonlinear error was less than 2.3% with an average seeds-mass-per-unit-area (SMA) of 0.3–2.4 g/cm^2^. Then, four sets of force sensors were installed symmetrically near the four corners of the vibrating tray which were used to measure the SMA respectively. Back propagation (BP) neural networks which take four SMA measurement results as input parameters were developed to monitor the total seed mass in the tray. Monitoring results using DEM simulation data showed that the general relative error was 3.0%. Experiments were carried out on a test-rig and the results validated that the relative error was reduced to 5.0% by using the BP neural network method.

## 1. Introduction

Rice is the most important food crop in China and has a planting area of about 30 million hectares. China produces more than 210 million tons of rice, which accounts for about 30% of the total world rice production [1]. Nursery-transplanting is the main cultivation technique since it produces strong seedlings and extends the growth period for more than half a month [2].

Nowadays, the hybrid-rice planting area is increasing sharply. Precision seeding has gradually become a major planting technique to improve the yield [3]. The shape of the rice seed is irregular with frangible glume on the surface. Vacuum seeders are widely used for rice precision seeding because they have many advantages compared with mechanical seeders such as more precise seeding quality, a lower rate of seed damage, better control and adjustment, and a broader spectrum of applicability [4,5,6,7]. As a kind of rice precision seeding equipment especially used in the nursery process, a vacuum plate seeder is primarily composed of a suction plate and a seed tray [8]. According to the structure of the seedling nursery tray, multiple nozzles are installed on the panel of the suction plate. These nozzles perform their work under the suction force of vacuum pressure and pick up the seeds from the tray, which are then held in the nozzles. The tray should be vibrated at high frequency and low amplitude so that the seeds in the tray can separate from each other. This reduces the interactive forces and allows for easy and precise seed pick up. The vertical distance between suction nozzles in the suction plate and the vibrating seeds is an important operating parameter [9]. To maintain the continuous working performance of the seeder, it is important to monitor the total seed mass in the seed tray in real time and accurately control the pickup position of the suction plate accordingly. The key problem for accomplishing this task is monitoring the total seed mass in the tray. However, under practical conditions, it is inevitable that there are some uncertain vibration interferences. This causes the seeds to flow in the tray resulting in a time-varying seed distribution, which greatly increases the difficulty of accurate measurement.

Motion characteristics and distribution states are the basis of total seed mass measurement. Computational techniques such as the discrete element method (DEM) are now commonly applied to probe the particle dynamics [10,11,12]. DEM simulations can provide more information, such as the trajectories of and transient forces acting on individual particles, which is extremely difficult to obtain by physical experimentation [13]. The overall system behavior is determined as a result of individual particle interactions. The application has been greatly expanded since the introduction of the multisphere method to describe nonspherical shapes. Many agricultural seeds such as rice, wheat, maize, and soybean have been represented successfully [14,15,16,17,18]. Therefore, DEM was used here to simulate the motion of seeds in a rectangular vibrating tray, and according to the obtained characteristics of the seeds’ vertical impact force, a measurement method for seed mass in a small area was proposed. Then, four sets of force sensors were used to monitor the total seed mass, and a back propagation (BP) neural network was developed to improve accuracy. Finally, experiments were carried out to evaluate the monitoring performance.

## 2. Materials

### 2.1. DEM Simulations

A commercial three-dimensional DEM code (EDEM^®^ 2.5, DEM Solutions) was used to perform the simulations. Particles were assumed isotropic and to have perfectly smooth bodies, and they were deemed to overlap to model the deformation of the contacting surfaces during impact. According to the physical properties of the rice seed, a triaxial ellipsoidal particle model was established using multiple spheres. The Hertz–Mindlin contact model was selected to calculate the interaction forces of seeds and the impact forces between seeds and the tray [19,20,21]. This model has been successfully used for various agricultural grains, and the normal force *F_n_* can be calculated by

(1) $$F_{n}=\frac{4}{3}E_{0}\delta_{n}^{\frac{3}{2}}\sqrt{R_{0}}-2\sqrt{\frac{5}{6}}\frac{\ln e}{\sqrt{\ln^{2}e+\pi^{2}}}\sqrt{2E_{0}}\sqrt[{4}]{R_{0}\delta_{n}}\sqrt{m_{0}}v_{n}^{rel}.$$

The tangential force increment Δ*F_τ_* corresponding to the incremental tangential displacement Δ*δ_τ_* can be expressed as

(2) $$\Delta F_{\tau }=8\delta_{n}G_{0}\theta_{k}\Delta \delta_{\tau }+{\left(-1\right)}^{k}\mu (1-\theta_{k})\Delta F_{n},$$

where *E*_0_ and *G*_0_ are the equivalent Young’s and shear modulus of the two interacting particles, *δ_n_* and *δ_τ_* are the normal and tangential overlap, *R*_0_ is the equivalent radius, *m*_0_ is the equivalent mass, *e* is the coefficient of restitution, *v_n_*^rel^ and *v_τ_*^rel^ are the normal and tangential relative velocity, *μ* is the friction coefficient, and *k* = 1, 2 and 3 correspond to the loading, unloading, and reloading processes. 

If |Δ*F_τ_*| < *μF_n_*, *θ_k_* = 1. Else if |Δ*F_τ_*| > *μF_n_*,

(3a) $$\theta_{k}={\left(1-\frac{F_{\tau }+\mu \Delta F_{n}}{\mu F_{n}}\right)}^{1/3},\;\Delta \delta_{\tau }>0,\;\mathrm{loading}$$

(3b) $$\theta_{k}={\left(1-\frac{F_{\tau }^{*}-F_{\tau }+2\mu \Delta F_{n}}{2\mu F_{n}}\right)}^{1/3},\;\Delta \delta_{\tau }<0,\;\mathrm{unloading}$$

(3c) $$\theta_{k}={\left(1-\frac{F_{\tau }-F_{\tau }^{**}+2\mu \Delta F_{n}}{2\mu F_{n}}\right)}^{1/3},\;\Delta \delta_{\tau }>0,\;\mathrm{reloading}.$$

The calculation procedure was to update the normal force *F_n_* and overlap *δ_n_*, followed by calculating the tangential force increment Δ*F_τ_*. The forces F_τ_^*^and F_τ_^**^ define the load reversal points and need to be continuously updated.

**Figure 1:**
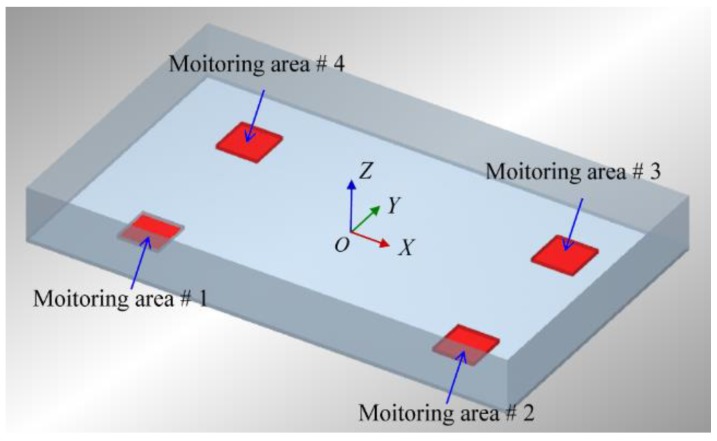
Schematic diagram of seed tray model.

The size of the established rectangular tray model in DEM simulations was 700 × 420 mm and the material selected was aluminum alloy #7075. The size and material of the model was the same as the actual seed tray, mainly to meet the requirements of seedling operation for a standard blanket nursery tray. The values of the material properties used in the DEM simulation are shown in Table 1. The center point of the tray was selected as the origin, and an inertial coordinate **X**_0_ = [*X Y Z*] was established (Figure 1). According to the preliminary experimental results, DEM simulations were carried out with the vibration frequency *f* of 11 Hz and an amplitude *A* of 4 mm. The average seed layer thickness was in the range of 15–25 mm [22]. According to the bulk density of rice seed, the calculated average seeds-mass-per-unit-area (SMA) *κ*_0_ was between 0.9 and 1.5 g/cm^2^.

In practical operations, some uncertain interference factors such as the precision of installation and the horizontal posture of the frame are inevitable, which usually leads to a small time-varying change in vibration direction. Therefore, the DEM simulation process was divided into two stages. In the first stage (0–10 s), the tray was sinusoidally vibrated in the vertical direction. The purpose was to analyze the vertical vibration characteristics of seeds and propose a potential measurement method of SMA. In the second stage (10−70 s), a continuous random time-varying rotational angle along the *X* and *Y* axes was applied to the vibration direction, which was used to simulate the distribution variation of seeds in the tray and analyze its influence on the total seed mass monitoring accuracy. Considering that the seed flow in the vibrating tray is also affected by the layer thickness, the average SMA *κ*_0_ was selected as 0.9, 1.2 and 1.5, respectively. The adjustment parameters are represented in Table 2.

### 2.2. Seeds Vibration Characteristics

**Figure 2:**
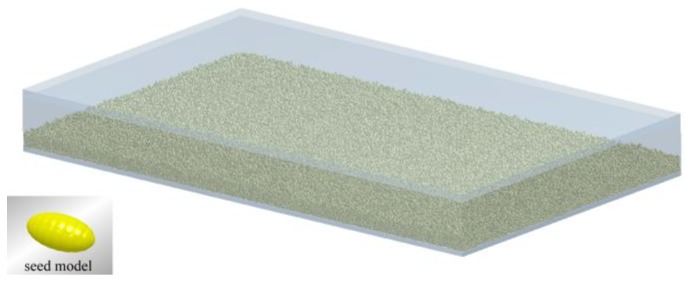
Snapshot of DEM simulation.

When the seed tray is sinusoidally vibrated in the vertical direction, the seeds are always in vibrational motion at their original locations and uniformly distributed in the tray, which is shown in Figure 2. The seeds are thrown up from the surface of tray and impact occurs due to the vibration strength *K*_v_ = *Aω*^2^/*g* being greater than 1. The motion reaches an approximate stable periodic state after about 10 vibration periods. Then, the parameters such as the vertical velocity of mass center *v*_Z_ and normal impact force *F_n_* action on the monitoring area are recorded.

**Figure 3:**
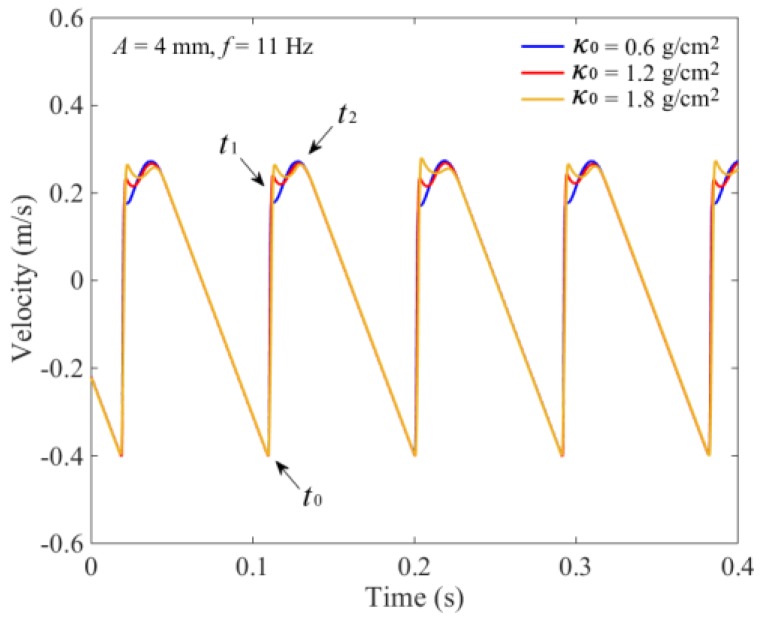
Variations of the vertical velocity of seeds’ mass center *v*_Z_.

Variations of the vertical velocity of the seeds’ mass center *v*_Z_ with different average SMA *κ*_0_ are shown in Figure 3. At the time *t*_0_, seeds begin to impact with the tray surface, and the *v*_Z_ increases quickly because seeds move synchronously with the tray. Seeds begin to be thrown up from the tray surface at time *t*_1_ and completely separate at time *t*_2_. In this process, the *v*_Z_ gradually increases to the maximum value. With the change of *κ*_0_, there is a certain fluctuation in *v*_Z_ which is majorly influenced by the internal interaction forces of the seeds. Then, under the action of gravity, the *v*_Z_ linearly decreases until the impact occurs again.

## 3. Methods

### 3.1. SMA Measurement Method

According to the impulse theorem, the change of seed momentum during the collision is equal to the impulse action on the plate. It can be found from Figure 3 that the difference in *v*_Z_ with different *κ*_0_ mainly occurred between times *t*_1_ and *t*_2_. The difference was small and could be ignored. Thus, the seeds’ mass could be measured by calculating the change of the seeds’ impulse during vibration. Using DEM simulation results of the normal impact force *F_n_* action on the tray surface, a second-order Butterworth low-pass filtering calculation was utilized to calculate the stable value of impulse *I* [20]. The critical frequency was 1 Hz, and the transmissibility equation was expressed as

(4) $$H(s)=\frac{A_{0}\omega_{c}^{2}}{s^{2}+\sqrt{2}\omega_{c}s+\omega_{c}^{2}},$$

where *ω_c_* is the critical angular frequency and *A*_0_ is the transmission gain.

**Figure 4:**
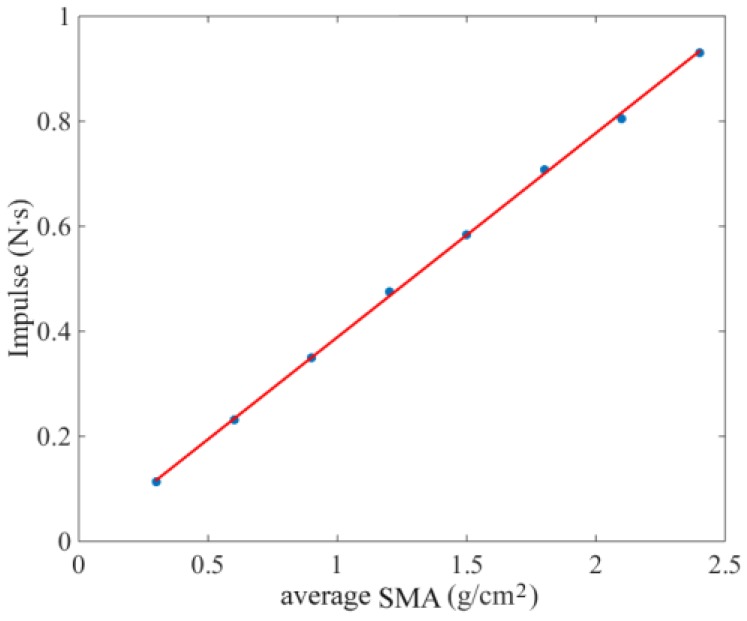
Linear relationship between *κ*_0_ and *I*.

With *κ*_0_ in the range of 0.3–2.4 g/cm^2^, the established linear relationship between *κ*_0_ and *I* is shown in Figure 4. The nonlinear fitting error was less than 1.8%. 

### 3.2. Monitoring Method Using BP Neural Network

**Figure 5:**
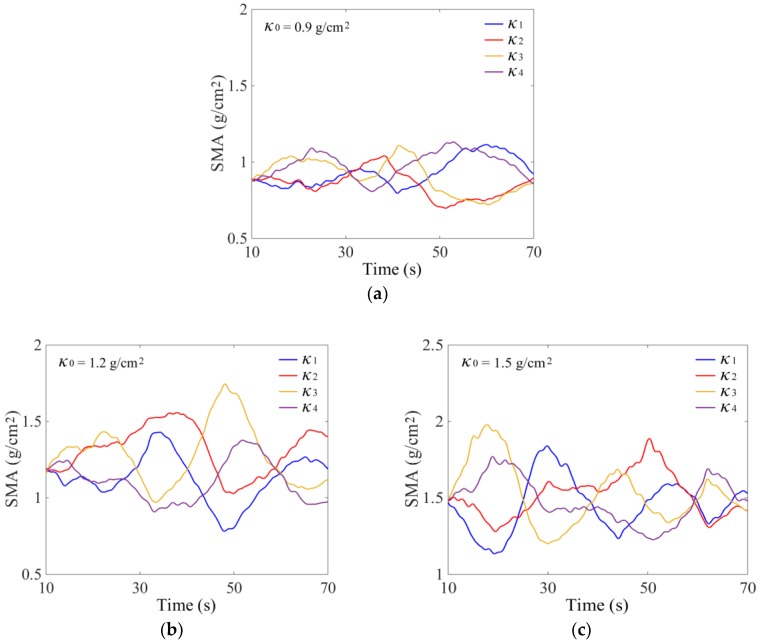
Variations of seeds-mass-per-unit-area (SMA) in four areas: (**a**) Parameters #1; (**b**) Parameters #2; and (**c**) Parameters #3.

The total seed mass in the tray *M* can be calculated as the product of average SMA *κ*_0_ and tray area *S*, *M* = *S* × *κ*_0_. The area *S* is a constant value for a certain tray. So, the problem is converted to the monitoring of *κ*_0_. Due to the shape of the rectangular seed tray, four 60 × 60 mm small square areas on the bottom were divided symmetrically near the four corners to measure the SMA, which is shown in Figure 1. The distances between the centers of each area and the corresponding vertex in the *X* and *Y* axes directions were 120 and 95 mm, respectively. Using the simulation parameters given in Table 2 and the proposed SMA measurement method, the calculated variations of SMA *κ*_1_, *κ*_2_, *κ*_3_, and *κ*_4_ in four areas are shown in Figure 5.

The seed motion under vibration excitation with interference is complicated and it is almost impossible to establish a definite mathematical model to describe the seed distribution variation. Moreover, neural network modelling is a new technology founded on the structure and operation of the human brain. It offers an alternate and powerful way to accurately model systems with variability and uncertainty. As a typical feed-forward neural network, the BP neural network is theoretically able to learn any nonlinear relationship at a desired level of precision by a form of supervised learning from representative datasets [23,24]. The algorithm error is sent back through the network to alter the weights to improve prediction accuracy. Here, in order to obtain the relationship between the *κ*_1_, *κ*_2_, *κ*_3_, and *κ*_4_ and the average SMA *κ*_0_, a three-layer BP neural network including an input layer, hidden layer, and output layer was established. The *κ*_1_, *κ*_2_, *κ*_3_, and *κ*_4_ were normalized on the interval [0, 1], and then used as the input of the BP neural network. The sigmoid function $g(x)=1/(1+\exp^{-x})$ was selected as the activation function of the hidden neurons, and the activation function of the output neurons was a linear function. The outputs at the hidden and output layers are given by

(5) $$h_{k}=g(\sum_{i=0}^{N}v_{ki}\kappa_{i}),i=0,1,\cdots ,N;k=1,2,\cdots ,Q,$$

(6) $$\kappa_{\mathrm{BP}}=\sum_{k=0}^{Q}w_{k}h_{k},$$

where *κ*_BP_ is the output of the network, *h*_k_ is the output of the *k*th hidden neuron, *κ_i_* is the *i*th input variable, *v_ki_* and *w_k_* are the connection weights from the inputs to the hidden layer and the hidden layer to the output layer, respectively, *N* = 4 is the number of input neurons, and *Q* is the number of neurons in the hidden layer. The BP algorithm error function was defined as $E=e^{2}/2={\left(\kappa_{0}-\kappa_{\mathrm{BP}}\right)}^{2}/2$, and the least mean square (LMS) algorithm was used to train the network to compute the weights *v_ki_* and *w_k_* that minimize overall error. The changes of the connection weights were calculated as

(7) $$\Delta v_{ki}=\eta \frac{\partial E}{\partial v_{ki}}=\eta h_{k}(1-h_{k})\kappa_{i}w_{k}e,i=0,1,\cdots ,N;k=1,2,\cdots ,Q,$$

(8) $$\Delta w_{k}=\eta \frac{\partial E}{\partial w_{k}}=\eta h_{k}e,$$

where *η* is the learning rate. To avoid oscillation or divergence and improve training speed, the learning rate *η* was selected as an exponential decay function, $\eta (t)=\eta_{0}\exp^{-t/\tau }$.

### 3.3. Structure of Vacuum Plate Seeder

**Figure 6:**
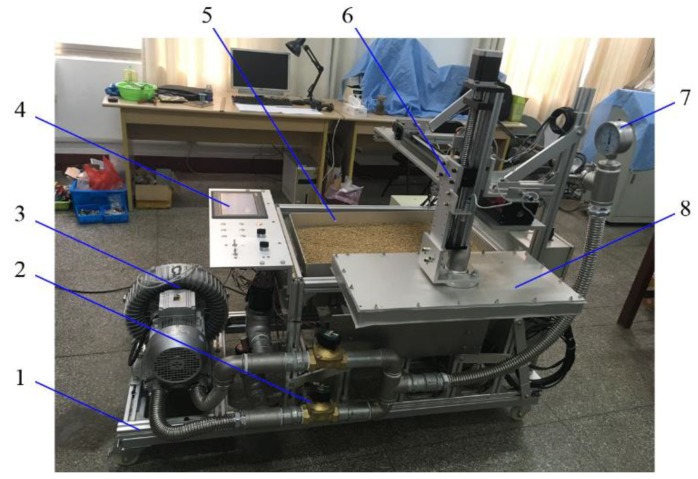
Vacuum plate seeder using rectangular vibrating tray: 1. Frame 2. Exchange valve 3. Fan 4. programmable logic controller (PLC) system 5. Seed tray 6. Cross-sliding table 7. Pressure gauge 8. Suction plate.

In this experiment, we designed a vacuum plate seeder in which a suction plate was installed on a cross-sliding table, which could perform horizontal and vertical motions. The differential pressure was generated by a fan, and the conversion between vacuum and positive pressure in the plate chamber was controlled by a set of exchange valves. Furthermore, the differential pressure could be adjusted by using a frequency converter to change the fan rotational speed. The pressure values were measured using a pressure gauge. The overall size of the rectangular seed tray was 700 × 420 mm. Driven by a motor and slider-crank mechanism, the tray reciprocated along a guiding axis. By adjusting a set of parallel mechanisms, the vibration direction could rotate along the *X* and *Y* axes with an angle of ±10°. The structure of the test-rig is shown in Figure 6.

According to the proposed SMA measurement method, cantilever force sensors were used to measure the seeds’ impact forces in four monitoring areas. One end of each sensor was fixed at the bottom of the tray, and a monitoring plate was fixed at the other end. The surface of the plate and seed tray was maintained at the same level. The capacity of force sensors was 500 g and the comprehensive error was 0.02%. Four sets of force sensors were installed on the tray, and the output voltages were recorded using a programmable logic controller (PLC) system.

### 3.4. SMA Measurement Device

**Figure 7:**
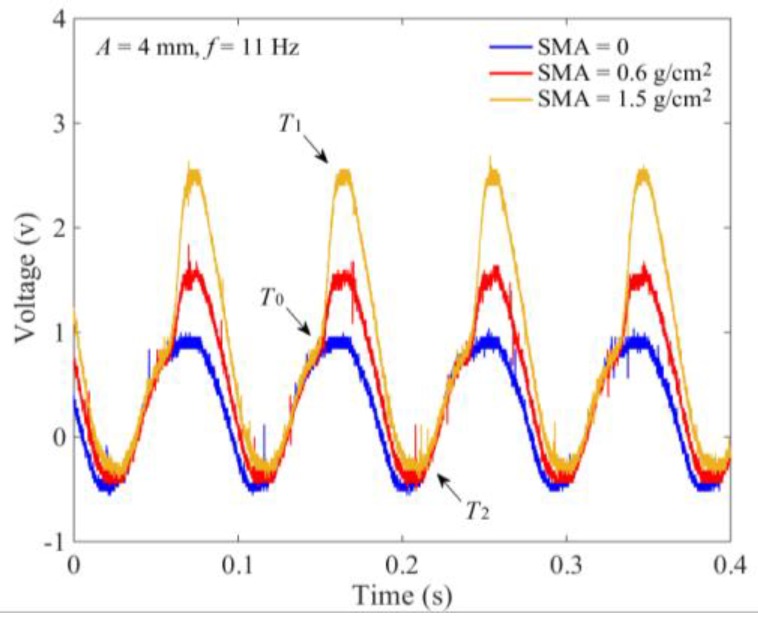
Output voltage of the cantilever force sensor at different SMAs.

When the tray was vibrated vertically with *f* of 11 Hz and *A* of 4 mm, Figure 7 shows the output voltage of a force sensor *V*_0_. Because the sensor was synchronously vibrated with the tray, the output signal *V*_0_ was generated by the inertial force of the monitoring plate with SMA of 0. In general, it was a sinusoidal periodic signal, although there were certain interferences. The amplitude mainly depended on the mass of the monitoring plate and the vibration strength. Influenced by the motion characteristics of the slider-crank driving mechanism and the installation accuracy, the vibration stability of the tray at the highest and lowest points was relatively low. At the phase angles of ±π/2, the fluctuation between vibration periods was relatively more substantial. Affected by the mass of the monitoring plate, there was an offset voltage in *V*_0_. When there were seeds in the monitoring area, the output signal *V*_0_ was coupled with the seeds’ impact force and the inertial force of the monitoring plate. Thus, to improve the weight of the seeds’ impact force, the monitoring plate was composed of an aluminum alloy material. The thickness and mass of the monitoring plate were 3 mm and 30 g, respectively. Furthermore, by comparing the *V*_0_ with SMA of 0.6 and 1.5 g/cm^2^, it was found that the changes of times corresponding to the initial impact *T*_0_ and the maximum force *T*_1_ were not noticeable. With the increasing of SMA, there was a slight extension of time *T*_2_ that seeds completely separated from the tray surface.

**Figure 8:**
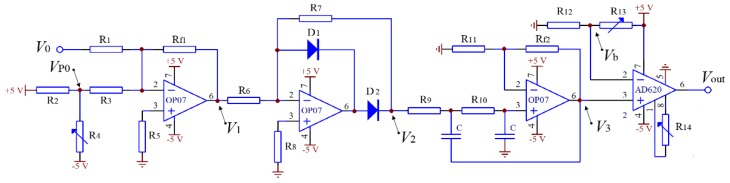
Signal process circuit.

According to the variation of *V*_0_, a signal process circuit for measuring SMA was designed, shown in Figure 8. Firstly, the *V*_0_ and an adjustable voltage *V*_P0_ were input to a reverse adder for the sum calculation to adjust the offset of voltage *V*_1_. Then, *V*_1_ was input to a precision half-wave rectifier to eliminate the negative signal of *V*_1_, so that the influence of vibration instability at the lowest point could be reduced. A Butterworth low-pass filter with a critical frequency of 0.5 Hz was applied to receive the stable mean value *V*_2_. Finally, the zero-point of the output voltage *V*_out_ and the measurement sensitivity were adjusted by changing the bias voltage *V*_b_ and gain of the differential amplifier. 

## 4. Results

### 4.1. DEM Simulation Data Analysis

**Figure 9:**
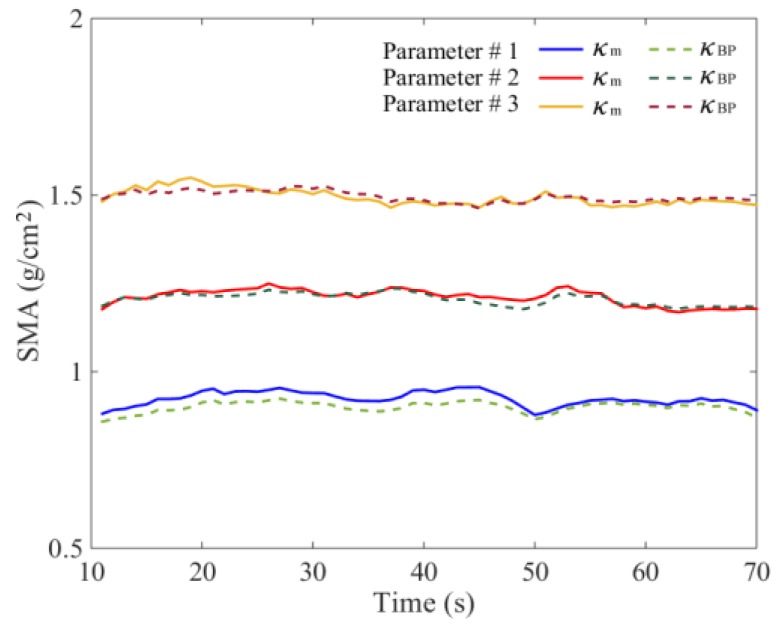
DEM simulation results of *κ*_0_ using *κ*_m_ and *κ*_BP_.

**Figure 10:**
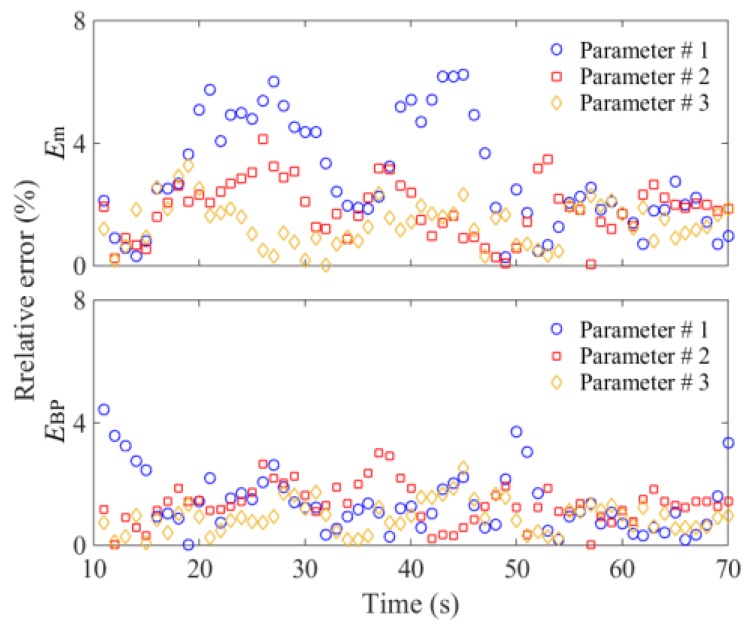
DEM simulation results of relative errors.

In order to verify the feasibility of the proposed method and determine the parameters of the BP neural network model, the total seed mass monitoring results were analyzed using the DEM simulation data. The *κ*_1_, *κ*_2_, *κ*_3_, and *κ*_4_ sampled with a frequency of 1 Hz were used as datasets. Randomly, 70% of datasets from three DEM simulations were selected respectively for training and the remaining datasets were used for testing the neural network model. It is essential to determine the number of neurons in the hidden layer. An inadequate number of hidden neurons will result in underfitting and cannot achieve the desired level of precision. Too many hidden neurons will result in overfitting and poor generalization. According to the recommended equation, the number of hidden neurons was tested from 6 to 15, and the initial learning rate *η*_0_ was 0.03 [25]. The results showed that the optimal error *E* was less than 3.5% with eight hidden neurons, and the accuracy would not improve when continually increasing the hidden neurons. Using *κ*_1_, *κ*_2_, *κ*_3_, and *κ*_4_ given in Figure 5, calculation results of *κ*_BP_ are shown in Figure 9. The general relative error using the BP neural network was less than 3.0% (Figure 10). We also used the datasets of Parameters #1 and 3 to train the model, and datasets of Parameters #2 for testing. The results indicated that the relative error also could be controlled near 3.0%, which proved that the established BP model had good generalization performance.

### 4.2. Monitoring Experiments

Calibration tests of the SMA measurement device were firstly carried out on the test-rig. The calibration range was extended to 0.3‒2.4 g/cm^2^ due to the reasonable range of average SMA *κ*_0_ usually ranging between 0.9 and 1.5 g/cm^2^ during the rice seeding operation. Linear least-squares fitting results showed that the relative nonlinear error was less than 2.3%.

**Figure 11:**
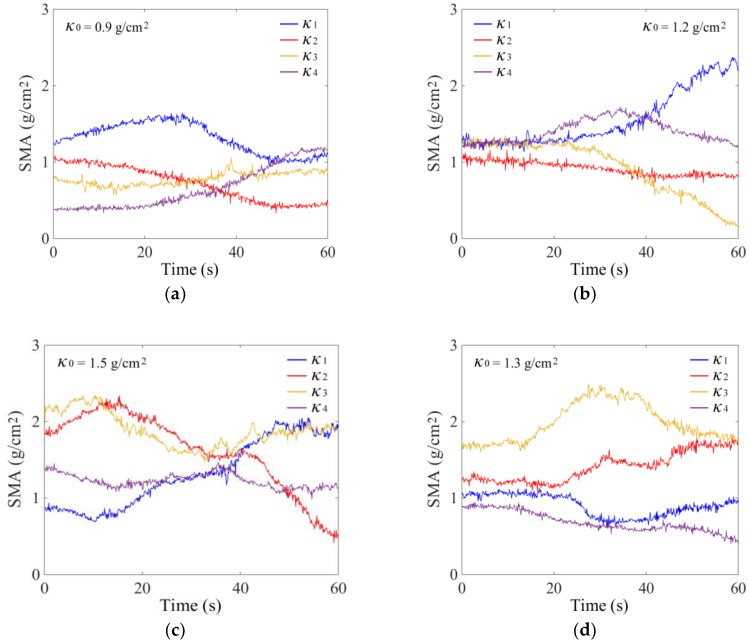
Experimental results of SMA in four monitoring areas: (**a**) *κ*_0_ = 0.9 g/cm^2^, (**b**) *κ*_0_ = 1.2 g/cm^2^, (**c**) *κ*_0_ = 1.5 g/cm^2^, and (**d**) *κ*_0_ = 1.3 g/cm^2^.

During the total seed mass monitoring experiments, a specified mass of rice seeds was laid evenly in the tray. The tray was vibrated with frequency *f* of 11 Hz and amplitude *A* of 4 mm. The vibration direction was manually adjusted to make the seed flow in the tray. With the average SMA *κ*_0_ of 0.9, 1.2, and 1.5 g/cm^2^, the measured variations of *κ*_1_, *κ*_2_, *κ*_3_, and *κ*_4_ are shown in Figure 11.

**Figure 12:**
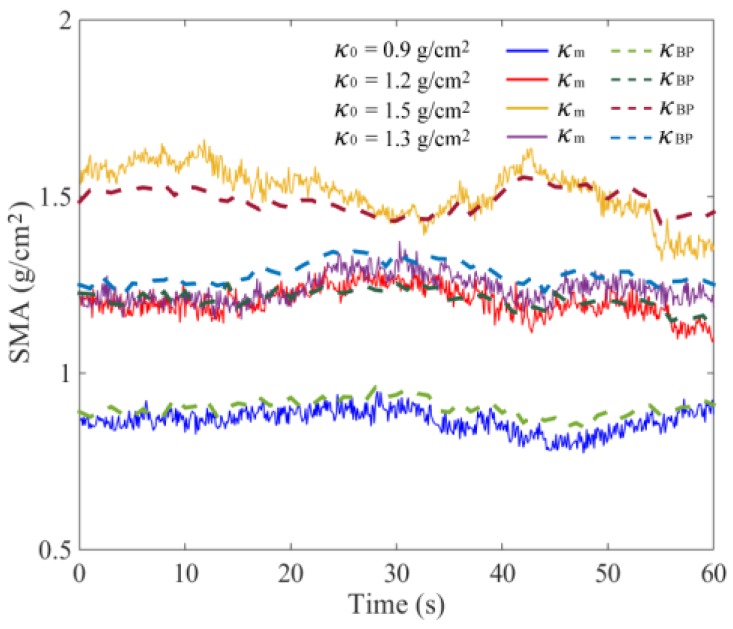
Monitoring results of *κ*_0_ using *κ*_m_ and *κ*_BP_.

**Figure 13:**
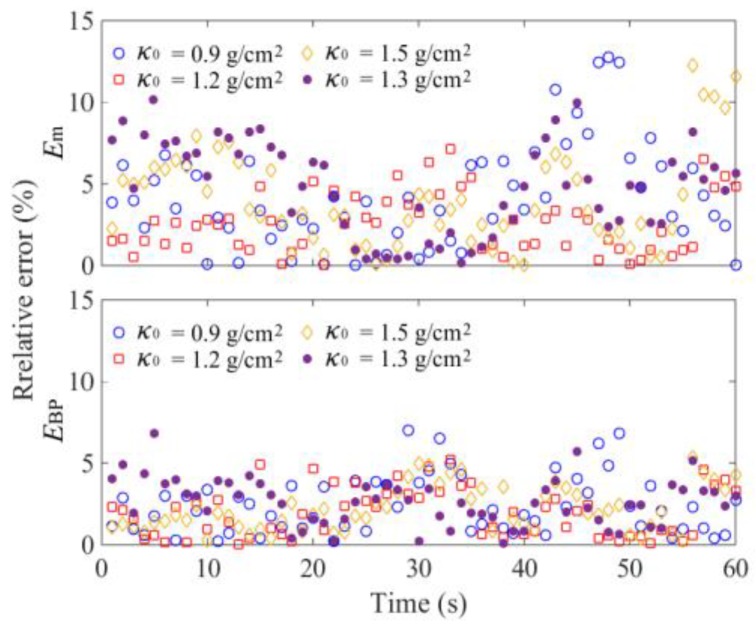
Experimental results of relative errors.

A BP neural network was established using the above method. After retraining, the received output results of *κ*_BP_ and the calculated relative errors *E*_BP_ are shown in Figure 12 and Figure 13. Influenced by sensor measurement error and actual, more complex interference factors, the actual monitoring error was slightly higher than the DEM simulation error. The value of *E*_BP_ was generally less than 5.0%. To further validate the generalization of the neural network, an additional experiment with *κ*_0_ of 1.3 g/cm^2^ was added. The measured variations of *κ*_1_, *κ*_2_, *κ*_3_, and *κ*_4_ are shown in Figure 11d, and the calculation results of relative error *E*_BP_ was generally less than 5.0%.

## 5. Discussion

The most common way to monitor the average SMA *κ*_0_ is to calculate the mean value of SMA *κ*_m_ in different areas, which can be expressed by

(9) $$\kappa_{m}=\frac{1}{4}(\kappa_{1}+\kappa_{2}+\kappa_{3}+\kappa_{4}).$$

The monitoring error of *κ*_m_ is greatly affected by the number of measurement areas. In practical situations, it is difficult to measure the SMA of several areas. Based on the DEM simulation data, when the tray was vibrated vertically (0–10 s), *κ*_m_ could well predict the variation of *κ*_0_ due to the even seed distribution in the tray. With *κ*_0_ of 0.9–1.5 g/cm^2^, the comprehensive nonlinear error was near 1.8%. When the interference was applied to the vibration direction (10–70 s), the calculated results of *κ*_m_ are shown in Figure 9. It was obvious that the relative error *E*_m_ between *κ*_m_ and *κ*_0_ was significantly increased. The maximum value of *E*_m_ was near 7.5%, which is shown in Figure 10. The most important reason for this phenomenon was the seed flow in the tray. The irregular and time-varying characteristics of seed distribution greatly increased the measurement error. The experimental results of the mean values of SMA *κ*_m_ and the relative error *E*_m_ are shown in Figure 12 and Figure 13, respectively. The overall errors were distributed within 10%, and the maximum value was near 14%. They were much larger than the BP neural network output errors.

The conclusion was drawn by comparing the measurement errors of the two methods. The internal relationship between the total seed mass and the distribution state could be well established using the BP neural network, and it is an effective way to improve monitoring accuracy. Furthermore, by using the measured *κ*_1_, *κ*_2_, *κ*_3_, and *κ*_4_, the seed distribution states in the vibrating tray also can be acquired [26].

## 6. Conclusions

In this paper, rice seed vibration and motion characteristics in a rectangular vibrating tray were simulated using DEM. Based on the analysis of vertical velocity variation of the seeds’ mass center, a monitoring method of seed mass in a small monitoring area was proposed. A signal processing circuit was designed that was mainly composed of a reverse adder, a precision half-wave rectifier, a low-pass filter, and a differential amplifier in series. Calibration experiments were carried out with a vibration frequency of 11 Hz and amplitude of 4 mm. The obtained results showed that the nonlinear relative error was less than 2.3% with SMA of 0.3–2.4 g/cm^2^.

Four sets of monitoring devices were installed near the four corners of the vibrating tray. The total seed mass in the tray was calculated by the mean values of the SMA in the four monitoring areas. However, the accuracy was severely affected by the seed motion and distribution states. A BP neural network was developed to monitor the average SMA, and the DEM simulation results indicated that the proposed method was a feasible method to improve monitoring accuracy. The experimental results proved that the monitoring relative error of total seed mass could be reduced to less than 5.0% with the average SMA of 0.9–1.5 g/cm^2^.

The proposed method could be used to monitor the total seed mass in real time and acquire the seed distribution states in the vibrating tray. This is an important basis for determining the pickup position of a suction plate and the design of an automatic control of a vibrating tray to uniformly discretize seeds, which would guarantee the continuous working performance of a vacuum plate seeder.

## Figures and Tables

**Table 1:** Values of material properties used in the discrete element method (DEM).

Parameter		Rice Seed	Tray
Semiaxes (mm)		2.85 × 1.55 × 1.35	/
Mass (g)		26.5 × 10^−3^	/
Density (kg/m^3^)		1080	2800
Young’s modulus (MPa)		375	72,000
Poisson’s ratio		0.25	0.33
Coefficient of friction	seed-seed	0.48	/
	seed-tray	/	0.32
Coefficient of restitution	seed-seed	0.42	/
	seed-tray	/	0.48
Time step (s)		1 × 10^−6^	

**Table 2:** Variations of the vibration direction in DEM simulations.

Time (s)	Parameter #1 (*κ*_0_ = 0.9 g/cm^2^)	Parameter #2 (*κ*_0_ = 1.2 g/ cm^2^)	Parameter #3 (*κ*_0_ = 1.5 g/ cm^2^)
	Direction Angles (°)	Direction Angles (°)	Direction Angles (°)
0–10	[90, 90, 0]	[90, 90, 0]	[90, 90, 0]
10–15	[88.7, 89.1, 1.51]	[89.7, 88.7, 1.50]	[88.6, 88.6, 2.00]
15–20	[91.2, 89.1, 1.50]	[89.2, 91.7, 1.94]	[90.1, 90.8, 0.73]
20–25	[89.6, 86.5, 0.52]	[91.6, 90.5,1.66]	[91.6, 91.8, 2.49]
25–30	[89.6, 91.5, 1.21]	[89.9, 91.8, 1.75]	[88.8, 91.0, 1.55]
30–35	[87.9, 90.1, 0.33]	[99.2, 92.1, 2.26]	[90.0, 89.0, 1.00]
35–40	[89.6, 87.5, 2.48]	[88.6, 98.1, 1.65]	[89.1, 89.4, 1.13]
40–45	[92.3, 90.5, 2.30]	[91.2, 91.5, 1.81]	[90.5, 91.3, 1.37]
45–50	[89.8, 89.9, 0.20]	[90.1, 91.0, 1.00]	[91.7, 89.7, 1.69]
50–55	[89.6, 91.0, 1.02]	[89.3, 91.4, 1.52]	[90.0, 89.0, 0.97]
55–60	[89.7, 90.0, 0.26]	[89.0, 90.6, 0.89]	[90.0, 87.5, 2.47]
60–65	[88.5, 90.0, 1.46]	[90.0, 89.4, 0.06]	[89.9, 91.5, 1.52]
65–70	[90.8, 89.9, 0.83]	[90.6, 89.0, 1.11]	[90.0, 89.5, 0.06]
[*α*, *β*, *γ*] are angles between the vibration direction vector and *X*, *Y*, and *Z* axes.			
